# Anatomical Variations in the Branching Pattern of Human Aortic Arch: A Cadaveric Study from Central India

**DOI:** 10.5402/2013/828969

**Published:** 2013-09-12

**Authors:** Virendra Budhiraja, Rakhi Rastogi, Vaishali Jain, Vishal Bankwar, Shiv Raghuwanshi

**Affiliations:** ^1^Department of Anatomy, L.N. Medical College, Kolar Road, Bhopal, India; ^2^Department of Biochemistry, L.N. Medical College, Kolar Road, Bhopal, India; ^3^Department of Preventive and Social Medicine, L.N. Medical College, Kolar Road, Bhopal, India; ^4^Department of Ear, Nose and Throat, L.N. Medical College, Kolar Road, Bhopal, India

## Abstract

Variations of the branches of aortic arch are due to alteration in the development of certain branchial arch arteries during embryonic period. Knowledge of these variations is important during aortic instrumentation, thoracic, and neck surgeries. In the present study we observed these variations in fifty-two cadavers from Indian populations. In thirty-three (63.5%) cadavers, the aortic arch showed classical branching pattern which includes brachiocephalic trunk, left common carotid artery, and left subclavian artery. In nineteen (36.5%) cadavers it showed variations in the branching pattern, which include the two branches, namely, left subclavian artery and a common trunk in 19.2% cases, four branches, namely, brachiocephalic trunk, left common carotid artery, left vertebral artery, and left subclavian artery in 15.3% cases, and the three branches, namely, common trunk, left vertebral artery, and left subclavian artery in 1.9% cases.

## 1. Introduction

Aortic arch (AA) is located in the superior mediastinum. In 65–80% of the cases the three branches arise from aortic arch, namely, the brachiocephalic trunk (BCT), the left common carotid artery (LCCA), and the left subclavian artery (LSA). The point of origin of BCT trunk lies to the right of midvertebral line and that of LCCA and LSA to the left of midvertebral line. Variations in the branching pattern of the AA range from differences in the distance between origins of different branches to the number of branches [1, 2]. The anatomical variations in the branching pattern of AA are significant for diagnostic and surgical procedures in the thorax and neck. The present study describes the AA branching pattern in cadavers from central India and discusses the findings according to their embryological and clinical implications.

## 2. Material and Method

The study was conducted on fifty-two cadavers at the Department of Anatomy, L.N. Medical College and research centre, Bhopal, India. The thoracic cavity was opened by cutting through the costochondral junctions and removing the sternum and costal cartilages. The lungs were removed, superior vena cava and brachiocephalic veins cleared, and pericardium opened to expose ascending aorta. Fibro fatty tissue and nerves were removed to clarify the branches of aortic arch and variations in branching pattern observed.

## 3. Results

**Figure 1:**
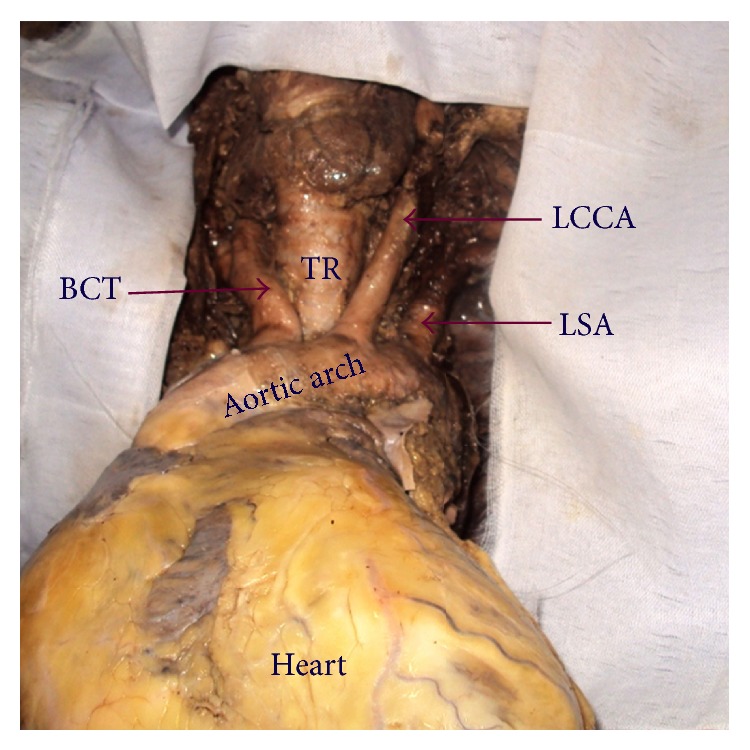
Aortic arch showing classical branching pattern. BCT: brachiocephalic trunk, LCCA: left common carotid artery, LSA: left subclavian artery, and TR: trachea.

**Figure 2:**
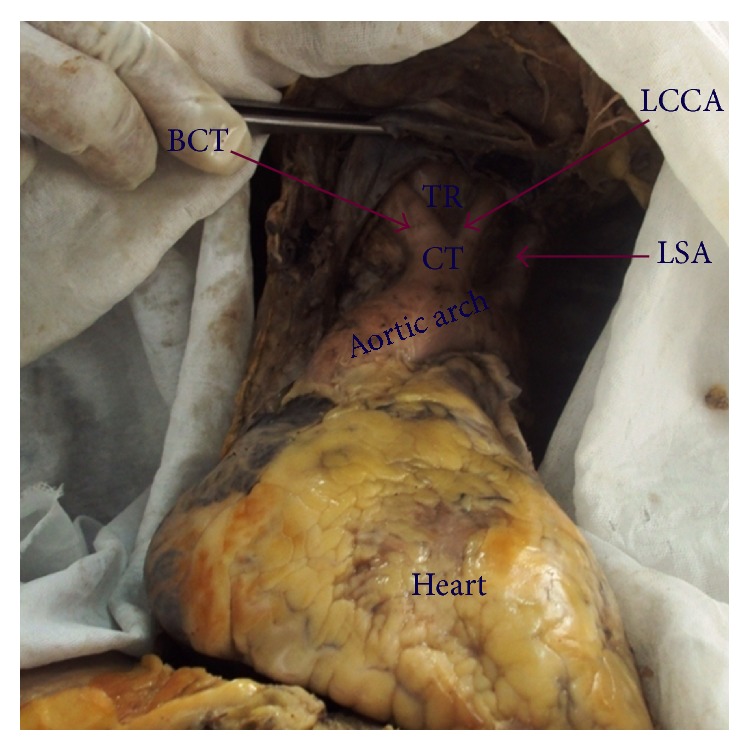
Aortic arch showing two branches (common trunk for brachiocephalic trunk and left common carotid artery). CT: common trunk, BCT: brachiocephalic trunk, LCCA: left common carotid artery, LSA: left subclavian artery, and TR: trachea.

**Figure 3:**
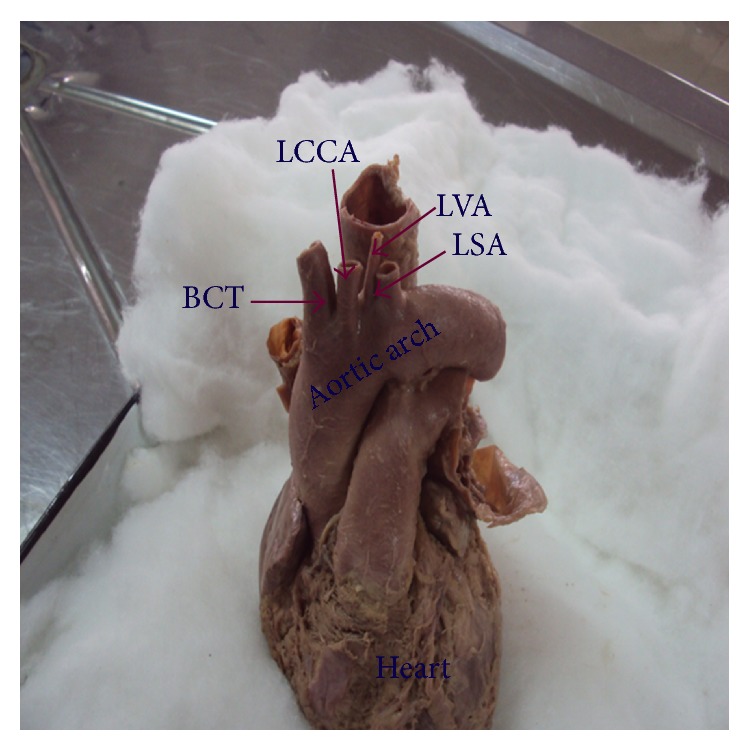
Aortic arch showing four branches. BCT: brachiocephalic trunk, LCCA: left common carotid artery, LVA: left vertebral artery, and LSA: left subclavian artery.

**Figure 4:**
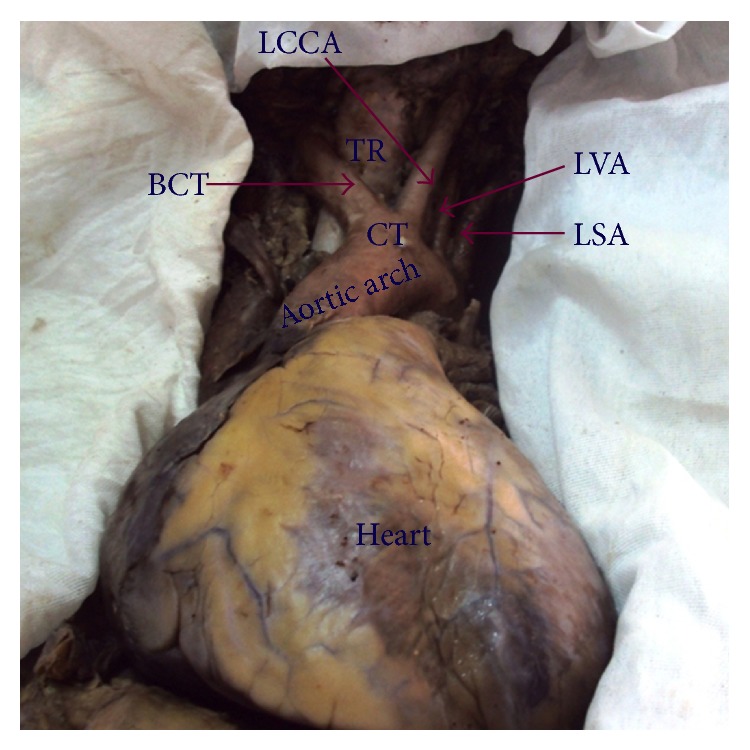
Aortic arch showing three branches (common trunk for brachiocephalic trunk and left common carotid artery). CT: common trunk, BCT: brachiocephalic trunk, LCCA: left common carotid artery, LSA: left subclavian artery, LVA: left vertebral artery, and TR: trachea.

**Figure 5:**
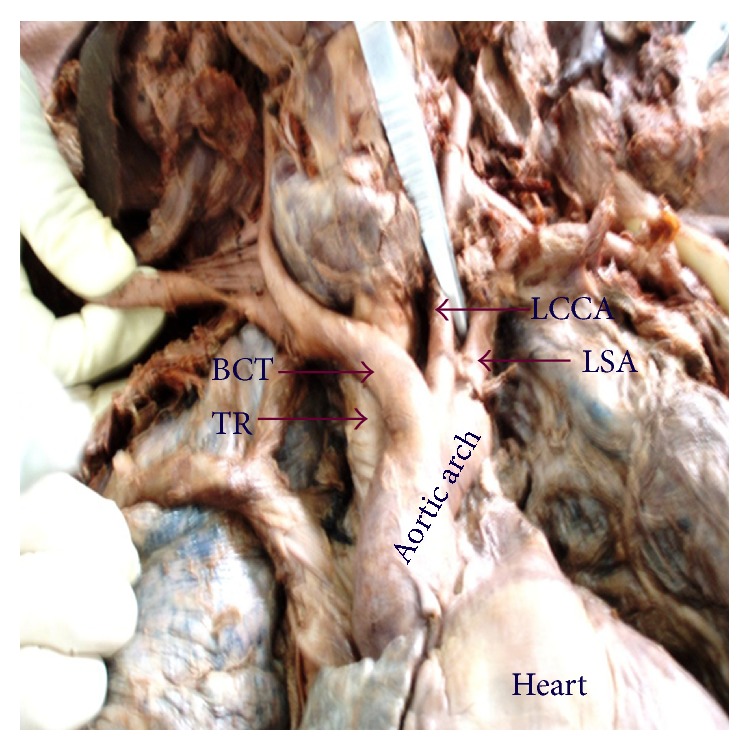
Point of origin of Brachiocephalic trunk lying left to midvertebral line. BCT: brachiocephalic trunk, LCCA: left common carotid artery, LSA: left subclavian artery, and TR: trachea.

In thirty-three (63.5%) cadavers the AA showed classical branching pattern of BCT, LCCA, and LSA (Figure 1). Nineteen (36.5%) cadavers showed variations in the branching pattern as ten (19.2%) cadavers had two branches, namely, LSA and a common trunk (CT) that gave origin to BCT and LCCA (Figure 2), eight (15.3%) cadavers had four branches, namely, BCT, LCCA, Left vertebral artery (LVA), and LSA (Figure 3), and one (1.9%) cadavers showed three branches, namely, CT, LVA, and LSA (Figure 4). The point of origin of BCT lies to the right of midvertebral line in fifty cases, but in two (3.8%) cases the point of origin was to the left of midvertebral line (Figure 5); here BCT crossed obliquely upward in front of trachea to reach from left to right side.

## 4. Discussion

The AA usually gives three branches, namely, the BCT, LCCA, and LSA. In the present study the usual three-branch pattern was observed only in 63.5% cases; however in 36.5% cases the aortic arch showed variations from usual branching pattern which was significantly higher when compared with previous studies involving different population groups (Table 1) [2–8].

The most common variant branching pattern which we observed in our study was the two-branch pattern. The two branches were the LSA and CT giving origin to BCT and LCCA. CT giving origin to BCT and LCCA which was previously reported by a number of authors in their case reports [15–17]. The results of the previous studies describing two-branch pattern in different population group varied from 1% to 28% as summarized in Table 2 [4, 5, 7–11].

Developmentally the two-branch pattern of the AA may be explained as follows. Aortic sac normally bifurcates into left and right limbs. Left limb of aortic sac forms the part of arch that intervenes between the origin of BCT and LCCA. If the aortic sac fails to bifurcate, then the LCCA will connect to aortic sac directly, resulting in bicarotid trunk or common trunk giving origin to BCT and LCCA as observed in 19.2% cases in our study [18, 19]. The approximation of LCCA to BCT is an important observation while invading the AA and its branches with instrument as all cases are susceptible to surgical attack [14, 20]. Nonrecognition of a critical AA at surgery may cause fatal consequences [5]. Sometimes such AA anomalies are clinically useful, as catheterization of LCCA originating from BCT or CT can be achieved without catheter exchange [9].

The next common pattern of branching of AA in our study was four-branche pattern. The four branches include BCT, LCCA, LVA, and LSA from right to left. The incidence of LVA taking origin from AA between origin of LCCA and LSA was significantly high in our study in comparison to previous studies in different population group (Table 3) [2, 4, 6, 10–14].

Developmentally the first part of LVA develops from proximal part of dorsal branch of seventh cervical segmental artery proximal to postcostal anastomosis. The second part is derived from longitudinal communications of the postcostal anastomosis. In the present study the left sixth segmental artery might have persisted as the first part of vertebral artery [21], or there is increased absorption of embryonic tissue of LSA between origins from the aortic arch to the origin of vertebral artery resulting in direct origin of the LVA from aortic arch [20].

The vertebral arteries arise from the superoposterior aspect of the first part of subclavian artery. The vessel takes a vertical posterior course to enter into the foramen transversarium of sixth cervical vertebra. The segment of the artery from its origin at subclavian artery to its respective transverse foramen is called the pretransverse or prevertebral segment [12]. The prevertebral segment of LVA of aortic origin is frequently affected by atherosclerosis [22]. Abnormal origin of vertebral artery may also favour cerebral disorder because of alterations in cerebral hemodynamic [23].

The third interesting finding in the present study was the occurrence of three branches, namely, CT, LVA, and LSA from right to left in 1.9% cases. Normally the point of origin of BCT lies to the right of midvertebral line; but in 3.8% cases we observed it to the left of midvertebral line, here BCT crossed obliquely upward in front of trachea to reach from left to right side. The shifting of BCT from right to left at origin may be explained as the cranial end of aortic sac drawn out into right and left limbs as the neck lengthens. The right limb becomes the BCT, and the left limb forms the part of definitive arch of aorta, which lies between the BCT and LCCA. By three years of age, growth of aortic arch causes the BCT to move cephaled, to the right and anterior away from trachea. In the present study in 3.8% cases the right limb of aortic sac deviates a little to the left of midline and to compensate for this abnormal origin of BCT takes an abnormal course [24]. Such anomalies of BCT are of vital significance during surgeries of throat and even more important in percutaneous dilatational tracheostomy, which has gained wide acceptance due to relative speed, simplicity, and ability to perform it on bedside as these variant anatomy may block the site for tracheostomy [25]. Knowledge of such variations of great vessels is of vital interest to the surgeons because a minor accidental injury of the vessels causes sudden massive hemorrhage [26].

## Figures and Tables

**Table 1:** Proportion of variant branching of aortic arch in different populations.

Author's name	Population	*N*	Percentage of aortic arch with variant branch pattern
Grande et al. (1995) [3]	Portuguese	33	18.0
Nelson and Sparks (2001) [4]	Japanese	193	5.7
Satyapal et al. (2003) [5]	South African	320	5.3
Gielecki et al. (2004) [6]	Polish	103	27.2
Shin et al. (2008) [2]	Korean	25	16.0
Natsis et al. (2009) [7]	Greek	633	17.0
Ogeng'o et al. (2010) [8]	Kenyan	113	32.7
Current study (2013)	Indian	52	36.5

**Table 2:** Incidence of two aortic arch branches in different populations.

Author's name	Population	*N*	Percentage of aortic arch with two branches (CT and LSA)
Nelson and Sparks (2001) [4]	Japanese	193	1.0
Satyapal et al. (2003) [5]	South African	320	3.4
Moskowitz and Topaz (2003) [9]	American	1480	3.2
Makhanya et al. (2004) [10]	South African	60	28.3
Natsis et al. (2009) [7]	Greek	633	15.0
Ogeng'o et al. (2010) [8]	Kenyan	113	25.7
Bhattarai and Poudel (2010) [11]	Nepalese	85	12.9
Current study (2013)	Indian	52	19.2

**Table 3:** Incidence of four aortic arch branches in different populations.

Author's name	Population	*N*	Percentage of four branches (BCT, LCCA, LVA, LSA)
Matula et al. (1997) [12]	Austrian	402	3.0
Voster et al. (1998) [13]	South African	60	5.0
Nelson and Sparks (2001) [4]	Japanese	193	4.1
Gielecki et al. (2004) [6]	Polish	103	6.8
Makhanya et al. (2004) [10]	South African	60	1.7
Bhatia et al. (2005) [14]	Australian	81	7.4
Shin et al. (2008) [2]	Korean	25	8.1
Bhattarai and Poudel (2010) [11]	Nepalese	85	7.0
Current study (2013)	Indian	52	15.3
